# Validation of the Arab Youth Mental Health scale as a screening tool for depression/anxiety in Lebanese children

**DOI:** 10.1186/1753-2000-5-9

**Published:** 2011-03-24

**Authors:** Ziyad Mahfoud, Sawsan Abdulrahim, Madeleine Badaro Taha, Trudy Harpham, Taghreed El Hajj, Jihad Makhoul, Rima Nakkash, Mayada Kanj, Rema Afifi

**Affiliations:** 1 Department of Public Health; Weill Cornell Medical College; Doha, Qatar; 2 Department of Health Promotion and Community Health; Faculty of Health Sciences; American University of Beirut; Lebanon; 3 Child and Adolescent Psychiatry; American University of Beirut Medical Center; Lebanon; 4 Department of Urban Development and Policy; London South Bank University; UK; 5 Department of Health Promotion and Community Health; Faculty of Health Sciences; American University of Beirut; Lebanon

## Abstract

**Background:**

Early detection of common mental disorders, such as depression and anxiety, among children and adolescents requires the use of validated, culturally sensitive, and developmentally appropriate screening instruments. The Arab region has a high proportion of youth, yet Arabic-language screening instruments for mental disorders among this age group are virtually absent.

**Methods:**

We carried out construct and clinical validation on the recently-developed Arab Youth Mental Health (AYMH) scale as a screening tool for depression/anxiety. The scale was administered with 10-14 year old children attending a social service center in Beirut, Lebanon (N = 153). The clinical assessment was conducted by a child and adolescent clinical psychiatrist employing the DSM IV criteria. We tested the scale's sensitivity, specificity, and internal consistency.

**Results:**

Scale scores were generally significantly associated with how participants responded to standard questions on health, mental health, and happiness, indicating good construct validity. The results revealed that the scale exhibited good internal consistency (Cronbach's alpha = 0.86) and specificity (79%). However, it exhibited moderate sensitivity for girls (71%) and poor sensitivity for boys (50%).

**Conclusions:**

The AYMH scale is useful as a screening tool for general mental health states and a valid screening instrument for common mental disorders among girls. It is not a valid instrument for detecting depression and anxiety among boys in an Arab culture.

## Background

Poor mental health in childhood and adolescence is a prevalent global public health challenge and accounts for a significant proportion of the disease burden and disability among young age groups worldwide [1,2]. Depression and anxiety are two common mental disorders (CMDs) [3], that have their onset in childhood or adolescence. As they are associated with a host of co-morbidities that carry into adulthood [4,5], early detection and adequate treatment of these disorders are pressing public health needs [6]. Yet, only a small proportion of children and adolescents with mental health conditions in general, and depression and anxiety specifically, are diagnosed in clinical settings and receive treatment [7,8]. More efforts are clearly needed to develop a community-based approach to detection and follow-up of CMDs [1]. This requires the development and validation of screening instruments that can be used as a first step in diagnosis.

Careful considerations should be given to measurement instruments that are both developmentally- and culturally-appropriate. Researchers and practitioners cannot assume that instruments developed for adult populations would capture the phenomena of depression and anxiety among young age groups. As such, a few of the most widely used screening instruments - such as the Center for Epidemiologic Studies Depression Scale, CES-D [9], and the General Health Questionnaire, GHQ-12 [10] - have been validated for use with children and adolescents. In addition to ensuring that a measurement tool is developmentally sound, the different ways in which CMDs are expressed cross-culturally should be taken into account [11]. Since conceptions of health and illness in general vary between cultural and linguistic groups, developing new instruments or adapting already existing ones for use in non-Western and non-English speaking countries is warranted.

The Arab countries in the Middle East and North Africa Region (MENA) have one of the largest proportions of youth compared to other world regions [12]. In 2005, around 21% of the total population in 19 Arab countries was comprised of those aged 15-24 years old. Countries in the MENA exhibit many of the factors that contribute to increased poor mental health among children and adolescents - namely political conflict and the rise of social disconnectedness with the expansion of low-income urban settings. Mental health services in urban centers are limited, of high cost, and unequally distributed [13]. Further, poverty and political conflict increase young people's exposure to negative major life events [14], which have been shown to increase the risk of mental distress and depression [15,16].

Research to explore the prevalence of CMDs among youth in the MENA and its associated burden is slowly gaining momentum. A review of mental health publications in the Arab world revealed that, between 1987 and 2002, there was an increase in mental health research in general and among children and adolescents specifically [17]. For example, whereas only one research study on the mental health of children and adolescents was published in 1991, a total of 12 were published in 2001. Recent evidence from Lebanon suggests the existence of high prevalence of mental disorders among the adult population coupled with an unmet need for detection and treatment [15,18]. Knowledge on the prevalence and burden of CMDs among children and adolescents in Lebanon is limited, highlighting the need for more community-based detection efforts that employ developmentally and culturally appropriate measurement instruments.

A review of mental health research in Arab countries [17] highlighted that most published studies were epidemiological and only a small proportion (8.6 percent of studies on children and adolescents) were psychometric in nature, i.e., designed to test the properties of a measurement instrument. The number of validated Arabic-language instruments to detect CMDs in adults as well as children and adolescents is very small. Only a few of the widely-used mental health scales have been adapted, translated, and validated for use with Arabic-speaking adults or children, such as the Edinburgh Postnatal Depression Scale [19], the TEMPS-A scale [20], and the Strengths and Difficulties Questionnaire, SDQ [21]. To our knowledge, only the SDQ was validated in Arabic among a youth population.

In this paper, we examined the validity and psychometric properties of the Arab Youth Mental Health (AYMH) scale as a screening tool for CMDs among Arabic-speaking youth. The AYMH scale was developed as part of a large community-based participatory intervention to improve the mental health of 10-14 year old children in a disadvantaged urban community in Beirut, Lebanon. Because ninth grade (age 14) was deemed by community partners as a critical period for youth, the intervention was planned to be administered prior to that age. As such, the evaluation instrument for the intervention, the AYMH scale, was developed to screen for CMDs among 10-14 year old children. The primary objectives of this paper were: 1) to examine the psychometric properties of the AYMH scale and 2) to validate the scale against a diagnostic assessment of depression and anxiety. The construct and clinical validation of the scale were carried out among 10-14 year old youth in Beirut, Lebanon.

## Methods

### Ethical Approval

Ethical approval for the study was obtained from the American University of Beirut's Institutional Review Board. The study protocol involved obtaining written consent from one of the parents of the child and a verbal assent from the child himself or herself. Recruitment was carried out by three trained social workers from a local Ministry of Social Affairs (MOSA) center through home visits. Participants who were determined to be in need of psychological counseling were referred to the American University Hospital child psychology clinic for up to 10 free visits.

### Sample

The sample consisted of 153 children between 10 and 14 years of age who were recruited through a convenience sampling strategy. The sampling frame consisted of all households with 10-14 year old children in a socioeconomically disadvantaged neighborhood serviced by the MOSA center. Inclusion criteria were any 10-14 year old child who was enrolled in school at the time of the study and who did not have any physical illness or disability. In cases where there was a child in the household who fit the inclusion criteria, a trained social worker explained to one or both parents the purpose of the study and sought their consent. To increase the sample size, social workers also recruited children who came to the MOSA center seeking a service from one of its social programs. In all cases, parents were informed that the study was carried out by university researchers and that a decision not to participate would in no way affect their ability to access services through the center.

### Screening Instrument and Diagnostic Assessment

#### Screening Instrument

The screening instrument for depression/anxiety consisted of the recently-developed AYMH scale in addition to a few demographic and wellbeing questions. The process of developing the scale for use in a community-based participatory intervention study has been described in detail in a recently published article [22]. In brief, the process of constructing the scale began with translating and reviewing a total of 14 English-language mental health measurement instruments that focus on CMDs and that have been previously used with youth. After soliciting community and professional opinion, researchers selected three for further consideration - the CES-D, the Hopkins Symptom Checklist, and the SDQ.

Focus group discussions were carried out with youth to test whether the mental health constructs in selected instruments were comprehensible and linguistically and culturally meaningful. Based on focus group results, researchers further examined and modified some constructs in the scales. To give a few examples, the researchers included items in the new scale that linguistically distinguish between feeling upset versus sad; added a new construct - feeling suffocated - because this expression was frequently invoked by youth during focus groups to express frustration; and changed the response options to include in addition to words a "star system," whereby a higher number of stars meant increasing intensity of experiencing a particular feeling. Based on this iterative process, the final scale was generated (see appendix 1).

It is worth noting that the scale was named an Arab Youth Mental Health scale, and not an anxiety/depression scale, to reflect the language employed by researchers and community members involved during the process of constructing it and throughout designing and implementing the intervention. The terms depression, anxiety, and disorder in Arabic, both linguistically and culturally, connote stigmatizing medical conditions. As such, the intervention was presented to community members, parents, and children as one designed to improve the mental health of children in general, so as not to imply erroneously that those who participate are admitting to having a mental disorder.

Data for the screening instrument were collected from children through an interviewer-administered structured questionnaire. This data collection step was carried out by a research assistant with a BA in psychology and in a private room in the MOSA center without interference from the child's parent or the psychiatrist. All items in the scale had a one-week recall period and were scored on a three-point Likert scale - rarely (one star), sometimes (two stars), and always (three stars). The range for the scale was 21 to 63, with a higher score indicative of poorer mental health. In addition to the scale items, the screening instrument collected data on age in five categories (9 &10, 11, 12, 13, 14 years old) and gender. It also included the self-rated health and self-rated mental health questions, both measured on a 5-point Likert scale (very good, good, fair, poor, very poor); due to sample size considerations, both variables were dichotomized in the analysis into very good, good, and fair versus poor and very poor. Finally, the instrument included a question on happiness (very happy, a little bit happy, not happy), worrying about the future (agree, not sure, disagree), and a question about enjoying life (agree, not sure, disagree).

#### Diagnostic Assessment

For the diagnostic assessment, a child and adolescent psychiatrist who was blinded to the results of the screening instrument conducted individual clinical interviews with each child participant, with at least one of his/her parents separately, and with both child and parent together to corroborate information. The presence and intensity of distressing signs and symptoms were evaluated and the Diagnostic and Statistical Manual of Mental Disorders, DSM-IV, criteria were employed to diagnose mental disorders. A symptom checklist covering all diagnostic categories was filled out, followed by an assessment of internalizing disorders using the Schedule for Affective Disorders and Schizophrenia (K-SADS) semi-structured questionnaire. In cases where there was suspicion of a disorder, the supplement for that disorder was filled out. The diagnostic interview also included ten minutes of unstructured assessment to evaluate the child's general wellbeing, school and family environment, stress, and trauma. A profile of each child was established along the five DSM-IV axes. All children diagnosed with a major depressive disorder, dysthymia, depressive disorder, or adjustment disorder with depressive mood were referred for psychiatric counseling. Similarly, all major anxiety disorders were considered positive diagnosis and the child was referred for psychiatric counseling. Given the AYMH scale's focus on CMDs, a diagnostic assessment of anxiety or depression by the psychiatrist was used as the standard reference to evaluate the specificity and sensitivity of the screening instrument.

### Statistical Analyses

Summary statistics using frequency distribution were used to describe the sample. Due to the small sample size in the youngest age group (n = 9), the 9- and 10-year old children were grouped into one category. The association between the scores on the mental health scale and other variables included in the instrument were evaluated using the t-test (for gender and psychiatric diagnoses of anxiety and depression) and one way analysis of variance (ANOVA) for associations with happiness, self-rated health, self-rated mental health, worrying about the future, and enjoying life, along with the Bonferroni's method for pair-wise comparisons when needed. We used Levene's test to check the equality of variance assumption.

Internal consistency of the scale was evaluated using Cronbach's alpha. As for validity analysis, the diagnostic assessment of depression and anxiety by the psychiatrist was used as standard reference. The Receiver Operator Curve method was used to determine the best cut-off for the scale, one that produced the best balance between sensitivity and specificity and the best agreement with the diagnostic assessment measured using the kappa statistic. All analyses were carried out for the total sample and for girls and boys separately using the Statistical Package for Social Sciences (SPSS, version 16, Chicago, USA). Significance levels were set at the 5% level.

## Results

The mean score on the AYMH scale (screening instrument) for the total sample was 34.63 with a standard deviation of 8.12. This mean score did not significantly differ by gender nor by age. Table 1 presents results of the ANOVA tests for differences in mean scores on the AYMH scale by the self-reported variables included in the screening instrument. These means were significantly associated with happiness, self-rated health, self-rated mental health, worrying about the future, and not enjoying life. The associations were in the expected direction whereby the mean scores on the AYMH showed a graded increase (poorer mental health) as adolescents reported less happiness, poorer self-rated health, poorer self-rated mental health, worrying about the future, and not enjoying life. Similar results were found for girls and boys with the exception of self-rated health (only significant among girls) and worrying about the future (only significant among boys).

**Table 1:** Comparisons of mean scores of AYMH scale by different variables

Variable	Total Sample			Girls		Boys	
	
	N(%)	Mean	p-value	Mean	p-value	Mean	p-value
Age			.250		.071		.520
9-10	40 (26.3)	33.85		34.74		33.05	
11	27 (17.8)	32.12		30.73		33.13	
12	41 (27.0)	36.33		37.73		35.45	
13	26 (17.1)	36.09		38.62		32.80	
14	18 (11.8)	35.06		31.43		37.36	

Gender			.472				
Boy	84 (54.9)	34.19					
Girl	69 (45.1)	35.17					

Happiness			<.001*		.004*		.008*
Too much	33 (21.6)	32.69 **A**		35.81 **AB**		29.56 **A**	
Happy	57 (37.3)	33.51 **A**		31.36 **A**		34.94 **AB**	
A little bit	52 (34.0)	35.20 **A**		36.43 **AB**		34.12 **AB**	
Not happy	11 (7.2)	43.28 **B**		46.25 **B**		41.57 **B**	

Self-rate health			.002*		.006*		.189
Very good	26 (17.0)	30.54 **A**		30.56 **A**		30.53	
Good	81 (52.9)	33.97 **AB**		33.17 **A**		34.48	
Fair	30 (19.6)	37.34 **B**		37.63 **AB**		37.00	
Poor/very poor	16 (10.5)	39.00 **B**		41.40 **B**		35.00	

Self-rated mental health			<.001*		<.001*		.009*
Very good	20 (13.1)	27.40 **A**		26.63 **A**		27.92 **A**	
Good	59 (38.6)	33.84 **B**		33.31 **AB**		34.28 **AB**	
Fair	46 (30.1)	35.67 **BC**		36.83 **BC**		34.79 **AB**	
Poor/very poor	28 (18.3)	40.04 **C**		41.84 **C**		38.36 **B**	

Worried/afraid about future			.010*		.358		.035*
Agree	95 (62.1)	35.92 **A**		36.05		35.81 **A**	
Not sure	30 (19.6)	34.40 **AB**		34.31		34.50 **AB**	
Disagree	28 (18.3)	30.56 **B**		31.17		30.38 **B**	

Not enjoying life			<.001*		<.001*		.010*
Agree	45 (29.4)	38.55 **A**		40.05 **A**		37.29 **A**	
Not sure	33 (51.0)	37.30 **A**		39.17 **A**		36.06 **AB**	
Disagree	75 (49.0)	31.16 **B**		30.76 **B**		31.50 **B**	

* Significant differences at the 5% level. Followed by Bonferroni's pairwise comparisons where similar letters indicate no difference between groups

Overall, 27 (17.6%) children were diagnosed with anxiety or depression. Significantly more girls than boys were diagnosed - 17 (24.6%) and 10 (11.9%), respectively. Internal consistency of the AYMH scale was good (Cronbach's alpha of .86) and did not differ between the two genders (Table 2). Considering the diagnostic assessment as the gold standard, the AYMH scale had moderate capabilities to discriminate between cases and non-cases of depression and anxiety for the total sample (Area under ROC curve = .71). However, the discriminatory capability of the scale was better for girls (Area under ROC curve = 0.78) than for boys (Area under ROC curve = 0.60). The cutoff 39/40 was the one that produced the best balance between sensitivity and specificity. This means that anyone who scored 40 or more on the scale was considered as a probable case for depression or anxiety. According to this cut-off point, sensitivity and specificity for the total sample were 63% and 79%, respectively. Although specificity remained the same for boys and girls, sensitivity was only 50% among the boys. Moreover, the mental health scale correlated well with diagnosed depression and anxiety in girls but not in boys. In particular, girls who were diagnosed with depression and anxiety scored on average significantly higher on the mental health scale as compared to those who were not diagnosed. The same trend was observed for the boys but it did not reach statistical significance (p = 0.10).

**Table 2:** Validity, sensitivity and specificity of the AYMH scale against clinical assessment for depression and anxiety

	Cronbach's Alpha	Area under ROC	Best cut-off value	Sensitivity	Specificity	Diagnosed AYMH mean score	Not diagnosed AYMH mean score	p-value
Total	.86	0.71	39/40	.63	.79	40.00 (9.08)	33.36 (7.40)	<.001*

Boys	.86	0.60	39/40	.50	.79	38.00 (11.26)	33.51 (7.41)	.100

Girls	.86	0.78	39/40	.71	.78	41.43 (7.27)	33.16(7.45)	<.001*

*Significant differences at the 5% level

## Discussion

Anxiety and depression are two of the most common mental disorders that often begin in childhood and adolescence. The detection and treatment of these two conditions in early developmental phases is imperative in a region that has a large proportion of youth and many of the factors that contribute to the onset of mental disorders. The main goal of the present validation was to contribute to the development of linguistically- and culturally-appropriate instruments for use in the early detection of CMDs in general, and anxiety and depression specifically, among Arab children and adolescents in the MENA region.

The validation revealed that the AYMH scale has reasonably good construct validity and internal consistency. However, the scale has moderate discriminatory capabilities as a diagnostic tool for depression and anxiety. Compared to a psychiatric assessment, the AYMH scale has low sensitivity and is a weak instrument to use as a diagnostic screening tool for depression and anxiety, especially among boys. The scale's ability to detect depression and anxiety is moderate for girls (70% sensitivity) and poor for boys (i.e., half of all boys diagnosed with depression or anxiety through a clinical psychiatric assessment were missed by the scale). By comparison, the SDQ showed better discriminating capabilities for psychiatric diagnoses when validated in Arabic [21], though it is important to note that the questionnaire was administered with the teachers and parents of children and not the children themselves.

The difference in diagnostic capability of the AYMH scale by gender deserves discussion. Research has consistently reported a higher prevalence of depression in women [23,24]. Findings of the studies we reviewed from the Arab region are consistent with those from international studies, showing that women and adolescent girls exhibit poorer mental health in general compared to men and adolescent boys, respectively [20,25]. In contrast, girls in the present validation did not significantly score higher than boys on the AYMH scale. Yet, the scale was moderately sensitive in detecting depression and anxiety for girls but not sensitive for boys. A potential explanation for this finding may lie in the nature of the items that make up the scale, namely that items may be biased towards detecting depression and anxiety among girls but not boys in an Arab culture. This corroborates with the body of literature which suggests that there is a "masculine" form of depression that is under-detected because it manifests through aggression and anger [26]. With respect to the AYMH scale, only one out of 21 items can be said to capture a form of aggressive behavior which captures a masculine expression of depression (item 15: fighting for no particular reason). Despite the rigorous process through which the scale was constructed, its inability to capture gendered feelings and behaviors indicative of CMDs meant that it missed half of the boys who were diagnosed with depression or anxiety by an experienced child and adolescent psychiatrist. In the future, we suggest that research focus on exploring gendered differences among Arab children and adolescents. With respect to the AYMH scale, we suggest incorporating items that capture externalizing behavior suggestive of mental disorders among boys.

Despite the poor sensitivity of the AYMH scale as a screening tool for depression and anxiety in boys, other robust psychometric properties of the scale merit its use as a screening tool for general mental health states in children and adolescents. Mean scores on the AYMH scale were associated with measures often employed to detect poor mental health states (such as single-item questions on happiness, self-rated health, and self-rated mental health). In general, adolescents who reported not being happy, being worried, and not enjoying life scored worse on the scale. Moreover, poor self-rated health (with the exception of the subsample of boys) and poor self-rated mental health were strongly associated with poor health. These findings and the good internal consistency of the scale suggest that the AYMH scale, though is not a good screening tool for depression and anxiety among boys, nonetheless measures mental health states and is a good tool to employ in community- and population-level screening efforts as a first step in detecting signs of CMDs among youth. The internal consistency of the scale is comparable to that observed for the CES-D scale (with a Cronbach's alpha of 0.82) when examined among American Indian adolescents [9].

It is important to acknowledge some of the limitations of the study. First, the sample was relatively small (153 children), which also meant that only a small number of children were diagnosed with depression and anxiety. Second, because participants were recruited through a social service center located in a disadvantaged community in Beirut, the validation findings may not be generalizable to Lebanese youth of different socioeconomic or regional backgrounds. Finally, the convenience sampling strategy might have biased our sample, whereby parents who felt a need for their child to undergo a mental health check up consented more than other parents and whereby compliant children agreed to participate more than others. Notwithstanding the limitations of the present validation and the low clinical validity of the AYMH scale among boys, we argue that the scale is still useful given its good internal psychometric characteristics. We recommend its use as a preliminary screening test for CMDs, with the important caveat to incorporate items on externalizing behavior in order for the scale to capture the gendered ways in which CMDs manifest among boys in an Arab culture.

Depression, anxiety, and mental states among Arab children and adolescents may be constructed and expressed differently than among youth in other cultures. With growing research interest in the MENA region to understand mental disorders and to measure their prevalence and risk factors, there is a clear need for more culturally adapted and validated scales for use among youth. The AYMH scale fills an important gap and addresses some of the limitations identified when examining some of the established instruments. The scale has gone through a rigorous process of development and is responsive to the context in which it was intended to be used. It uses simple language and specific terms which are commonly exchanged among Arab youth. We argue that even though the AYMH scale has limited use as a screening tool for depression and anxiety among boys, it has other positive attributes to justify its future use as a first step in screening for poor mental health states in 10-14 year old children.

## Competing interests

The authors declare that they have no competing interests.

## Authors' contributions

ZM participated in the design of the study, carried out statistical analysis, and drafted the methods and results. SA participated in the design and drafted the manuscript. MB and TEH carried out data collection. RA, JM, and RN participated in the design and coordination of data collection. TH provided feedback on drafts of the manuscript. All authors read and approved the final manuscript.

## Appendix 1: The Arab Youth Mental Health Scale

1. During the last week I was upset

2. During the last week I burst into tears several times

3. During the last week I was feeling scared and frightened

4. During the last week I felt suffocated

5. During the last week my sleep was interrupted because I was thinking of so many things

6. During the last week I was tense and nervous

7. During the last week I felt lonely

8. During the last week I was sad

9. During the last week I was worried

10. During the last week I was having difficulty concentrating on what I was doing

11. During the last week I felt dizzy/light headed

12. During the last week I didn't feel like talking

13. During the last week I was bored and I hated my life

14. During the last week I didn't have any hope for the future

15. During the last week I was fighting for no particular reason

16. During the last week I was bored and I had nothing to do

17. During the last week I was having thoughts of death

18. During the last week I was feeling emotionally drained

19. During the last week my heart was beating fast even without doing any type of sports

20. During the last week I was feeling fidgety and moving a lot. I couldn't sit still for a long time without any particular reason

21. During the last week, I was having a lot of headaches, stomach-aches, and nausea
